# The evolutionary history of mitochondrial porins

**DOI:** 10.1186/1471-2148-7-31

**Published:** 2007-02-28

**Authors:** Matthew J Young, Denice C Bay, Georg Hausner, Deborah A Court

**Affiliations:** 1 Department of Microbiology, University of Manitoba, Winnipeg, MB, R3T 2N2 Canada

## Abstract

**Background:**

Mitochondrial porins, or voltage-dependent anion-selective channels (VDAC) allow the passage of small molecules across the mitochondrial outer membrane, and are involved in complex interactions regulating organellar and cellular metabolism. Numerous organisms possess multiple porin isoforms, and initial studies indicated an intriguing evolutionary history for these proteins and the genes that encode them.

**Results:**

In this work, the wealth of recent sequence information was used to perform a comprehensive analysis of the evolutionary history of mitochondrial porins. Fungal porin sequences were well represented, and newly-released sequences from stramenopiles, alveolates, and seed and flowering plants were analyzed. A combination of Neighbour-Joining and Bayesian methods was used to determine phylogenetic relationships among the proteins. The aligned sequences were also used to reassess the validity of previously described eukaryotic porin motifs and to search for signature sequences characteristic of VDACs from plants, animals and fungi. Secondary structure predictions were performed on the aligned VDAC primary sequences and were used to evaluate the sites of intron insertion in a representative set of the corresponding VDAC genes.

**Conclusion:**

Our phylogenetic analysis clearly shows that paralogs have appeared several times during the evolution of VDACs from the plants, metazoans, and even the fungi, suggesting that there are no "ancient" paralogs within the gene family. Sequence motifs characteristic of the members of the crown groups of organisms were identified. Secondary structure predictions suggest a common 16 β-strand framework for the transmembrane arrangement of all porin isoforms. The GLK (and homologous or analogous motifs) and the eukaryotic porin motifs in the four representative Chordates tend to be in exons that appear to have changed little during the evolution of these metazoans. In fact there is phase correlation among the introns in these genes. Finally, our preliminary data support the notion that introns usually do not interrupt structural protein motifs, namely the predicted β-strands. These observations concur with the concept of exon shuffling, wherein exons encode structural modules of proteins and the loss and gain of introns and the shuffling of exons via recombination events contribute to the complexity of modern day proteomes.

## Background

Mitochondrial porins were first identified in paramecia, as proteins capable of forming voltage-dependent, anion-selective channels (VDAC) when inserted in artificial "black lipid" bilayers [1]. Proteins that formed pores with very similar characteristics were subsequently identified in mitochondria from fungi, plants, metazoans and invertebrates (See Table 1 for references), initially suggesting that mitochondria harbour a single form of porin. All of these proteins were of similar size (28–36 kDa) and formed anion-selective pores with conductances of about 4 nanoSeimens (nS) in artificial bilayers. Application of voltage, in the order of 50 mV, across the membrane converted the pores to a partially closed (1–2 nS), cation-selective state (voltage-dependent gating, reviewed by [2]). The biological relevance of the gating process is not clear, but it presumably reflects common types of voltage-sensitive interactions among segments of the proteins that contribute to both pore size and ion selectivity.

**Table 1:** Characteristics of mitochondrial porin isoforms.

Organism	*Porin isoform*	Pore size (nS)^a,b^	Ion selectivity^c^	Gating	Complements Δ*por1 *yeast	Reference
*Drosophila melanogaster*	DVDAC or porin (AAL47980.1)	4.1^d ^4.5^e^	anion^d,e^	yes^d,e^	yes	[65]
	CG17137 or Porin2-PA (NP_609462)	(variable) 0.5–8^d ^4.5^e^	variable^d ^cation^e^	variable^d ^no^e^	yes	[65^d^, 89^e^]
	CG17140 (AAF53019)	1.38	anion	required 110 mV^f^	no	[65]
	CG17139 (NP_609462)	none	n/d^g^	n/d	no	[65]
*Saccharomyces cerevisiae*	Por1p or VDAC1 (NP_014343)	4.1	anion	yes	n/a^h^	[90, 91]
	Por2p or YVDAC2 (NP_012152)	none	n/d	n/d	yes – at high copy	[39]
*Mus musculus*	VDAC1 (Q60932)	4.3	anion	yes	yes	[92]
	VDAC2 (NP_03582)	3.8	anion	yes	yes	[92]
	VDAC3 (NP_35826)	highly variable	n/d	n/d	partial	[92]
*Homo sapiens*	VDAC1 (NP_003365)	4.1	anion	yes	yes^i^	[93]
	VDAC2 (NP_003366)	4.0	anion	yes	yes	[93]
	VDAC3 (NP_005653)	n/d	n/d	n/d	n/d	
*Triticum aestivum*	VDAC1 (P46274)	3.8^d ^– 4.1	anion	yes	yes	[94, 95]
	VDAC2 (S59546)	4.0^d^	anion	yes	yes	[94]
	VDAC3 (S59547)	4.0^d^	anion	yes	partial	[94]
*Lotus japonicus*	VDAC1.1 (AAQ87019)	n/d	n/d	n/d	yes	[40]
	VDAC1.2 (AAQ87020)	n/d	n/d	n/d	yes	[40]
	VDAC2.1 (AAQ87022)	n/d	n/d	n/d	yes	[40]
	VDAC3.1 (AAQ87023)	n/d	n/d	n/d	yes	[40]
	VDAC1.3 (AAQ87021)	n/d	n/d	n/d	no	[40]

^a ^unless otherwise stated, prepared from mitochondrial membranes

^b ^in 1 M KCl or 1 M NaCl

^c ^pAnion/pCation = 1.5–1.8

^d ^isolated following heterologous expression in *Saccharomyces*

^e ^recombinant *D. melanogaster *VDAC2, expressed in *E. coli *and refolded in 1% Genapol X-080 in the presence of 0.5% (w/v) cholesterol

^f ^the midpoint for gating is usually around 50 mV

^g ^n/d, not determined

^h ^n/a, not applicable

^i ^Complementation was performed with chimeric constructs in which the HVDAC1 and HVDAC2 genes were fused to the 11^th ^and 22^nd ^codons, respectively, of the yeast *POR1 *gene [93].

**Figure 1:**
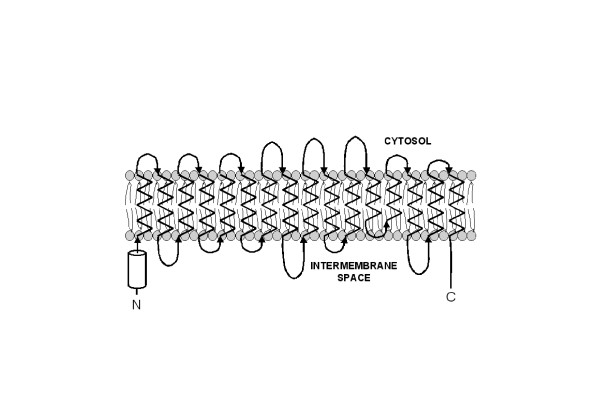
Overview of the predicted transmembrane arrangement of the *Neurospora *mitochondrial porin across the mitochondrial outer membrane. The model takes into account several secondary structure predictions, and experimental probing of the structure in artificial bilayers through the use of point mutations [14], deletions [9, 15, 16], and site-specific biotinylation [13], as described in [16]. The predicted N-terminal α-helix is represented by a cylinder, and putative β-strands by zig-zag lines, and loops and β-turns by curved arrows. The arrowheads point in the direction of the C-terminus of the protein.

The similar functional characteristics of mitochondrial porins suggest a common structure. These proteins presumably traverse the outer membrane as a series of β-strands that form a β-barrel, in a manner reminiscent of bacterial porins (Fig. 1; reviewed by [2-4]). A β-barrel pore was initially predicted from primary sequence analysis, which revealed the absence of potential membrane-spanning helices [5,6]. This observation has held for all mitochondrial porins known to date, and has been supported by biophysical analyses that reveal high β-strand content in liposome-embedded or detergent-solubilized porins [7-10]. Numerous approaches, including secondary structure predictions [11,12], and characterization of modified porins [13,14] or deletion variants [9,15,16] in artificial bilayers have led to predictions of porin topology, but a precise structural model has remained elusive (reviewed in [4]). Presumably there is a great deal of flexibility in the sequences that can comprise the β-strands of the barrel, as the primary sequence identity among porins from different species is low.

Porins are the most abundant proteins in the mitochondrial outer membrane (for example see [17]). The obvious function for these molecules is the exchange of ions and small molecules, including NADH [18], and ATP [19], across the mitochondrial outer membrane (reviewed by [20]). Regulated transport of these key metabolites has been proposed to control mitochondrial and therefore cellular energy transactions. Further studies have implicated porins in more complex roles, driven by interactions of VDAC with mitochondrial (for examples see [21,22]) and cytosolic (see [23-26]) proteins, and perhaps components of the cytoskeleton [27,28]. Given its general importance to cell biology, it is not surprising that links between disease and VDAC have been documented. One of the most intriguing roles of porin is its participation in the initiation of apoptosis. VDAC, the ADP/ATP carrier of the inner membrane, and cyclophilin D comprise the large permeability transition pore (PTP, [29,30]). Interactions of VDAC with pro and anti-apoptotic members of the Bcl-2 family including Bax [31-33], Bid [34], and Bcl-XL [35] have been proposed to regulate cytochrome *c *release via different mechanisms involving VDAC opening [31,32] or closure (reviewed in [36]). Finally, cytochrome *c *release from porin-containing vesicles lacking Bcl-family proteins has been demonstrated, suggesting that porin oligomerization can be responsible for PTP formation [37]. Regardless of the precise mechanism(s) of PTP regulation, it is clear that mitochondrial porin is engaged in complex interactions driving many facets of cell function.

This complex picture of porin function has been complicated in the last ten years by the identification of multiple porin isoforms in many cell types. Heins *et al. *[38] first isolated two porins from potato mitochondria (POM34 and POM36), both of which form pores in black lipid bilayers. In *Saccharomyces*, a genetic screen revealed a second isoform that, in high copy number, complemented a strain in which the known VDAC gene, *POR1 *was disrupted [39]. Advances in molecular techniques, and the accumulation of large amounts of sequence data from genome sequence projects and expressed sequence tag (EST) libraries, have led to an abundance of information regarding porin isoforms (see [Additional File 1], Table 1 and references therein]. The number of porin variants ranges from a single isoform in fungi such as *Neurospora*, to perhaps five in *Lotus japonicus *[40], although in this case some of the gene sequences were obtained from a cDNA library and therefore may represent different alleles at the same locus. In the few cases where they have been performed, electrophysiological analyses and complementation of the yeast *Δpor1 *strain (see Table 1) have revealed that each organism expresses at least one porin isoform with the characteristics of the originally described VDAC; this functional conservation is even more striking given the relatively low levels of primary sequence conservation (see below). In contrast, some porin variants are not capable of forming characteristic channels. Specialization of porin function is suggested by developmental regulation or tissue-specific differential expression of human [41], mouse [42] and plant [43,44] porin isoforms. Further evidence comes from cell lines and knock-out mice lacking porin isoforms singly or in combination [45].

Generally, the genes encoding porin isoforms are located on different chromosomes (for example, in mice [46]. An exception is *Drosophila *spp., where they are arranged in tandem repeats [47]. The evolutionary history of some of these multiple forms has been evaluated through phylogenetic analyses of the primary sequences of mitochondrial porins. These studies utilized the relatively low number of porin sequences available (< 60) and were focussed on animals [48] or plants [40,44]. It was revealed that porins from plants, animals and fungi form distinct groups that mirror the 16S rRNA phylogeny of these organisms [44]. Three clades were observed in vertebrates; each corresponding to the VDAC1, VDAC2 and VDAC3 groupings described for mammals [48]. Five porin subfamilies were identified in plants [40], some in only monocots or dicots, and others were represented in both groups.

The goal of this work is to use the large amounts of genome sequence data currently available to perform a comprehensive analysis of the evolutionary history of mitochondrial porins. In particular, more fungal porin sequences, which were under-represented in previously published phylogenetic analyses, were utilized. In total, 244 VDAC protein sequences, including newly-released sequences from stramenopiles, alveolates, and seed and flowering plants were analyzed. A combination of Neighbour-Joining and Bayesian methods was used to determine phylogenetic relationships among the proteins. The aligned sequences were also used to reassess the validity of the eukaryotic porin signature motif (Prosite PS00558), whose universality has already been questioned [40], and to search for signature sequences characteristic of VDACs from plants, animals and fungi. Finally, secondary structure predictions were performed on the aligned VDAC primary sequences, revealing a remarkable conservation in β-strand forming regions in spite of low sequence similarity in these segments. These structural predictions were used to evaluate the sites of intron insertion in a representative set of the corresponding VDAC genes.

## Results and Discussion

### Phylogenetic history of eukaryotic porins

**Figure 2:**
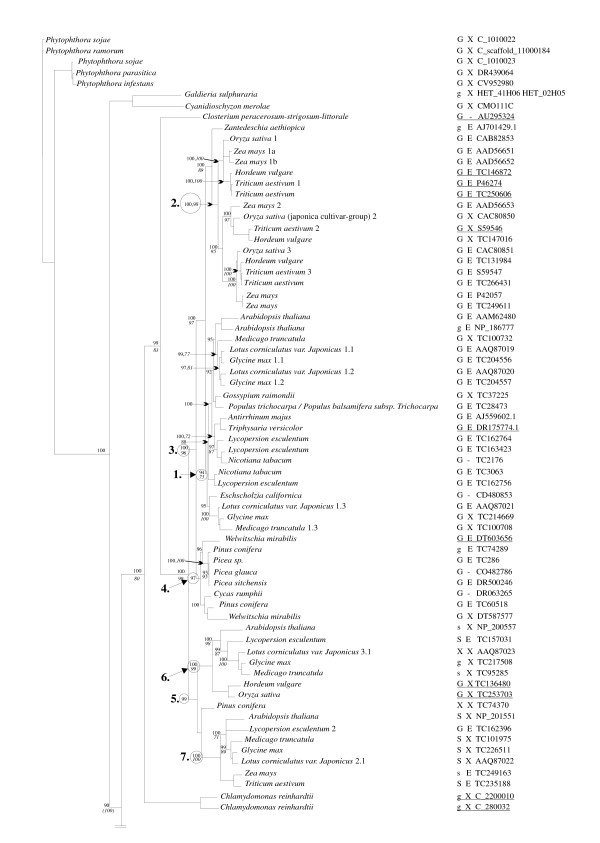
Evolutionary history of eukaryotic porin sequences – stramenopiles and plants. The phylogenetic tree, continued in Figure 3, is based on NJ and Bayesian analysis of 141 VDAC sequences. Stramenopile sequences were used as the out group. Levels of confidence of the nodes are only provided if support is above 66%. The numbers are based on posterior probability values generated by Bayesian analysis and on bootstrap analysis in combination with NJ analysis (*italics*). The presence of the GLK (G) and Eukaryotic porin motifs (E) are indicated towards the right of the phylogenetic tree. Note among the plants the GLK domain appears as the STK (S) motif; an X indicates the absence of the motif. Lower case g designates a G-any-K or G-any-R, where "any" refers to any other amino acid. The lower case s indicates S-any-K or S-any-R. The minus (-) indicates that the sequence was incomplete and thus the GLK and eukaryotic porin motifs could not be identified. Nodes designated by a number (1–10) are discussed within the text. Underlined accession numbers are those of VDACs that do not contain the signature motif identified in this study.

**Figure 3:**
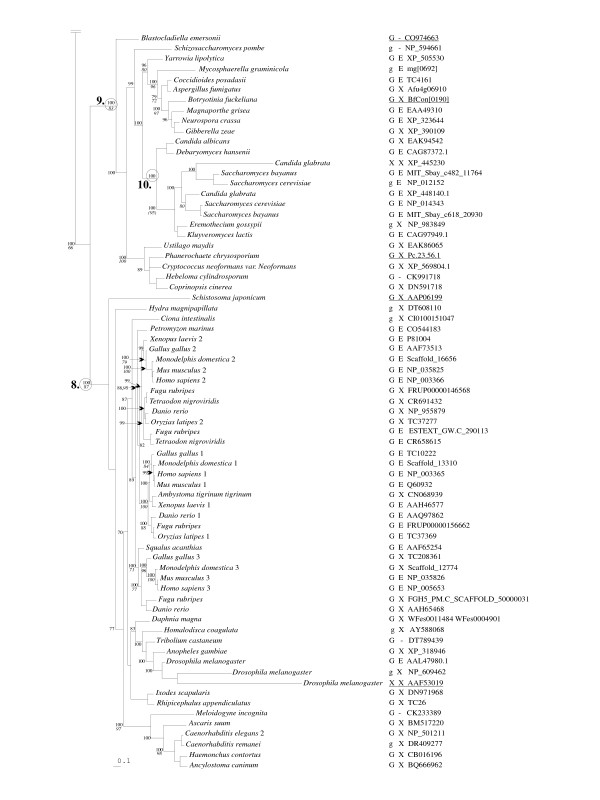
Evolutionary history of eukaryotic porin sequences – fungi and animals. The portion of the phylogenetic tree, described in Figure 2, containing the fungal and animal data is presented. Symbols and notations are as described for Figure 2.

Phylogenetic trees were obtained based on analysis of the aligned data set and subsets thereof with NJ and Bayesian algorithms. The phylogenetic estimates presented in Figs. 2 and 3 and [Additional File 2] were analyzed with NJ and Figs. 2 and 3 also include the results obtained with Bayesian analysis. The aligned data set was built on the smaller sets used by others [40,44,48] and includes VDACs from three eukaryotic crown groups (Plants, Animals, Fungi) and from the Stramenopiles. The latter included only five taxa (representing the Oomycota) and the use of *Phytophthora sojae *porin sequences as outgroups in our analysis is in part justified as the emergence of the Stramenopiles is viewed as a basal event to the evolution of the plants, metazoans and fungi [49]. A single representative of the Charophyta and two from the Rhodophyta were also used [see Additional File 1].

The VDAC amino acid sequences from members of the three crown groups formed monophyletic groupings and the branching patterns suggest that the animal and fungal porins are derived from a common ancestor. All methods of analysis yielded phylogenetic trees that were essentially congruent with each other. Essentially the evolution of the VDAC sequences follows the expected pattern for a highly conserved sequence, as the positions of the crown group taxa within the tree correspond in part to the expected phyletic positions based on rDNA sequences, which suggests that fungi and the metazoans share a common ancestor [49]. Our results place the stramenopile VDACs as outliers to the three main clades. The salient features of each group and subgroups within them are discussed below.

#### Plant mitochondrial porins: Angiosperms, Eudicots

Among the Eudicots, mitochondrial porins appear to have a rather complex evolutionary history. Based on the available data, there are potentially up to five paralogs for some members of this group of flowering plants, including *Glycine max *and *Lotus japonicus*. In cases where whole genome data are unavailable, it is not clear whether these represent allelic variants or different loci. Functional data have not been obtained for many of these isoforms, but in *Lotus japonicus*, all five forms are expressed throughout the plant, with only slight differences in expression levels in the tissues tested [40].

Many eudicot VDACs can be derived from one node (Fig. 2, node 1); thereafter the phylogenetic tree suggests that several gene duplications have occurred, giving rise to some of the recent eudicot VDAC paralogs. The current annotation for plant VDACs is very confusing and needs to be addressed in future efforts. Knowing the evolutionary history of the various VDACs, combined with the completion of more plant genome projects in the near future should facilitate this goal.

#### Plant mitochondrial porins: Angiosperms, Monocots

Within the monocots grouped by node 2 (Fig. 2), three clades can be recognized that suggest that at least three VDAC paralogs have evolved. The three monocot VDAC paralogs can be derived from a node that received 100% Bayesian posterior probability support and 89% bootstrap support in NJ analysis, suggesting these three paralogs have a common ancestor.

#### Plant mitochondrial porins: Gymnosperms

Basal to node 3 (Fig. 2) that joins the monocot and eudicot VDACs described above, a branch emerges that unites VDACs from the Gymnosperms (Seed Plants, node 4, Fig. 2). Although there were only a limited number of sequences available, there is an indication that more than one form of mitochondrial porin is present within some members (*Welwitschia mirabilis*, and *Pinus conifera*), suggesting that VDAC paralogs also evolved within this group of plants.

#### A clade composed of both seed and flowering plant porins

Attached to a node (Fig. 2, node 5) that is "basal" to the Angiosperm and Gymnosperm VDACs described above is a clade that includes mitochondrial porins from both monocots and eudicots, and the one available example of a seed plant VDAC. This clade could represent the most ancient forms of VDAC that were present in the common ancestor that gave rise to the seed and flowering plants. Again within this clade there appears to be evidence of paralogs, as more than one form of VDAC from *Arabidopsis thaliana*, *Lotus sp*., *Glycine max*, *Medicago sp*., and *Lycospersion sp*. are grouped within this cluster. This clade includes two groupings (nodes 6 and 7) that accommodate the latter paralogs. Also potentially allied to each of these two groupings are monocot sequences. The *Pinus conifera *porin sequence appears to be situated between the two groupings.

Overall within the land plants examined, VDAC paralogs appear to have evolved numerous times independently within different plant lineages. But ultimately one node appears to unite all the land plant porin sequences. Green algal sequences (Chlorophyta), and a Desmid sequence (Charophyta) are basal to the land plant node.

#### Metazoan mitochondrial porins

The evolution of metazoan VDAC genes (see node 8, Fig. 3) has already been addressed by Saccone *et al. *[48] and others. Essentially within the Chordates, the porin gene family consists of three genes (VDAC 1, 2, and 3) and these appear to be a classic example of a set of paralogous genes. Isoforms in the VDAC1 and VDAC2 clades are more closely related to each other than to those in the VDAC3 group [48]. For the basal branching members of the Phylum Chordata: tunicates (*Molgula tectiformis*) and the Urochordata (*Ciona intestinalis*), only one porin gene could be identified [see Additional File 1]. Thus, the Chordate porin paralogs probably evolved within the vertebrate lineage. In general, in the large data set (244 VDAC sequences), nodes basal to VDAC1 and VDAC2 vertebrate porin clades are poorly resolved (i.e. poor statistical support) and sequences representing the VDAC3 clade are placed next to sequences representing the amphioxus *Branchiostoma floridae *(Class Cephalochordata) and *Lymnaea stagnalis *(Pond Snail). In both data sets (141 and 244), the vertebrate VDAC3 clade appears to contain the earliest branching porin sequences. For *Petromyzon marinus *(parasitic marine lamprey) one of the oldest known taxa of living vertebrates (Hyperoartia), evidence for more than one porin gene was not recovered, although the entire genome sequence is not yet available.

Overall the species groupings were consistent with the current model of Metazoan evolution [50], with the echinoderms [*Strongylocentrotus purpuratus *(purple sea urchin)], cnidarians/hydrozoans [*Hydra magnipapillata *(fresh water polyp)], and members of the mollusk phylum, *Argopecten irradians *and *Spisula solidissima *(*i.e. *surf clam and scallop respectively) appearing basal to the vertebrate lineage of VDAC paralogs (Fig. 3 and [Additional File 2]). Although the branches placing members of the Phylum Mollusca received only poor statistical support [Additional File 2], one has to be cautious as the mollusk sequences were retrieved from EST data bases and may contain errors.

Among the invertebrates, as reported by Saccone *et al. *[48], and Graham and Craigen [47] only one VDAC gene could be recovered. However, within the Phylum Arthropoda, *Drosophila melanogaster *appears to have at least four porin genes ([Additional File 2], see nodes 1 and 2), of which one set might represent co-orthologs (see node 2), the result of a lineage specific duplication event (see [47]). However, a porin gene from *D. pseudoobscura *is derived from the same node that includes a potential set of co-orthologs ([Additional File 2], see node 2) and these *D. pseudoobscura *VDACs could represent inparalogs [51]. The two species of *Drosophila *(*melanogaster *and *pseudoobscura*) appear to have VDACs with the phylogenetic pattern expected for true paralogs; one paralog was then tandemly duplicated twice in *D. melanogaster *and once in *D. pseudoobscura*.

#### Fungal mitochondrial porins

Within the mycota the porin sequence phylogeny (node 9, Fig. 3) follows the expected pattern, with the Chytrid sequence being the basal member of this group, the Ascomycota and Basdiomycota sequences forming monophyletic groupings and the ascomycetous yeast branching early within the ascomycete lineage. With respect to gene duplication events, it appears that among the Saccharomycetales a lineage is present that displays the presence of paralogs (node 10, Fig. 3, and node 3 [Additional File 2]). *Candida glabrata*, and the *Saccharomyces spp. *appear to have at least two forms of the VDAC gene. The putative VDAC2 of *C. glabrata *is highly degenerate and is not present in the cluster of *Saccharomyces *VDAC2 sequences. It is of interest that we could not detect paralogs for the other yeast-like fungi, including *Eremothecium gossypii*, for which the entire genome has been sequenced. Recent genomic work on *S. cerevisiae *and allied species suggest that these organisms experienced genome duplication events during their evolution [52]. It is interesting to speculate that the appearance of VDAC paralogs coincides with this genome duplication event, and that for some unknown reason both copies of the porin gene were maintained.

### Phylogenetic history of porin paralogs

One question we tried to address in this analysis was the origin of VDAC paralogs. Did VDAC gene duplications arise early in the evolution of the eukaryotes or did gene duplications occur independently in different evolutionary lineages? If the first scenario applies it would suggest that VDAC duplication events in part paralleled the requirement for more specialized forms of VDACs as eukaryotes evolved into more complex multicellular or multi-tissue forms. Examples of such "ancient paralogs" are the genes for elongation factors involved in translation [53], and to a lesser extent the globin gene paralogs of the metazoans [54]. Alternatively, VDAC paralogs may have evolved independently in the different eukaryotic lineages.

Paralogs can arise through gene duplication, which in turn can be result of genome duplication events (polyploidy), segmental chromosome duplication as a result of unequal crossover events, or gene duplication events as a result of "retrotransposition" events. Recent comparative genomic analysis suggest that all of the above have occurred during the evolution of the eukaryotic crown groups, and several examples are revealed by the analysis of VDAC genes. For example, the presence of two versions of VDAC in *Saccharomyces*, but not other *Saccharomycetales *fungi such as *Kluyveromyces lactis *(Fig. 3), is in agreement with the genome duplication that is postulated for *Saccharomyces *[52]. Segmental duplication is seen in *D. melanogaster *and *D. pseudoobscura*, and appears to have led to highly divergent forms of VDAC [47]. Evidence for retrotransposition has not been seen; in the limited data set available, paralogs both lacking and containing introns have not been identified (see following discussion of VDAC gene structure).

Paralogs and orthologs can have different evolutionary fates. Specialization of porin function is suggested by developmental regulation or tissue-specific differential expression of human [41], mouse [42] and plant [43,44] porin isoforms. Further evidence comes from cell lines and knock-out mice lacking porin isoforms singly or in combination. For example, embryonic stem cell lines lacking any one of VDAC1, VDAC2 or VDAC3 display a 30% reduction in oxygen consumption, but only in the ΔVDAC3 cells is cytochrome oxidase activity at normal levels [45]. VDAC3^-/- ^mice appear normal, but are male-sterile due to sperm immobility, indicating a specific role for this isoform in mammalian reproduction [55]. In contrast, lack of VDAC1 is associated with *in utero *lethality, but interestingly, the surviving VDAC1^-/- ^offspring appear normal [56].

### Secondary structure predictions

**Figure 4:**
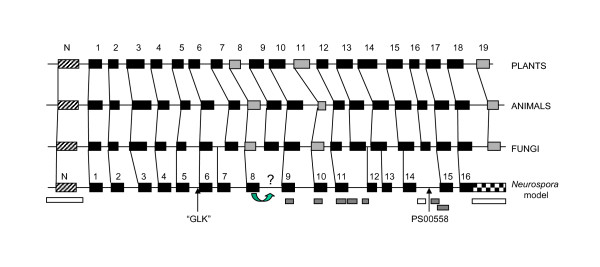
Predicted secondary structure elements in VDAC from the crown groups of plants, animals, and fungi. Predictions were made as described in Methods. For each summary diagram, the putative N-terminal α-helix is indicated by a hatched bar labelled "N" on the left, subsequent β-strands are indicated by filled rectangles, and the intervening loops are shown as thin lines. According to the model in Fig. 1, the N-terminal helix resides in the intermembrane space, and the subsequent loops and turns alternate between exposure to the cytosol and to the intermembrane space. β-strands with weak support are indicated in grey. The lower panel shows the model for *Neurospora crassa *VDAC structure derived in [16]. Structural elements are as described for the plant, animal and fungal models, except that the checkerboard region indicates a C-terminal segment that is exposed to the cytosol rather than forming the 19^th^β-strand (see text for discussion). Below the model of *N. crassa *VDAC structure, rectangles indicate segments, that when absent in porin variants, create molecules that form pores of wild-type conductivity (open), or inefficiently form pores that are either unstable or of reduced conductivity (filled) in artificial membranes. The position of the GLK sequence and the eukaryotic porin signature motif (PS00558) are also noted. Vertical lines connect regions of homology and the curved arrow indicates the discrepancy for the placement of β8 between previous models (see [4]) and the current predictions.

Although the precise transmembrane topology of mitochondrial porin from any source is not known, secondary structural predictions are useful in determining whether there have been constraints on sequence features, and therefore likely the structure of mitochondrial porins during their evolution. To this end, SSPRO was used to predict the secondary structure porin sequences aligned by PRALINE (see Methods). These predictions revealed remarkably similar patterns for all of the porins in this study. As shown in Fig. 4, a segment usually located less than 10 residues from the amino-terminus is the only region of the protein predicted to have α-helical character. The primary sequences of these putative α-helices are highly conserved within the major phylogenetic groups, and as discussed below may contain the best candidates for eukaryotic porin signature motifs. An intermembrane-space location for the N-terminal α-helix has been established through several experimental approaches [57,58].

In all cases, the remainder of the protein is predicted to be rich in β-strand, in agreement with existing models of porin structure (reviewed in [2-4,13,16,59]). Nineteen regions with β-strand propensity are predicted in the majority of porin sequences, these are numbered 1–19 in Fig. 4 and for simplicity will be referred to as β1, β2 etc. for the subsequent discussion. Remarkably, each of these regions corresponds to an aligned segment in the multiple sequence analysis, although the spacing between individual putative β-strands varies between the major phylogenetic groups (Fig. 4).

**Figure 5:**
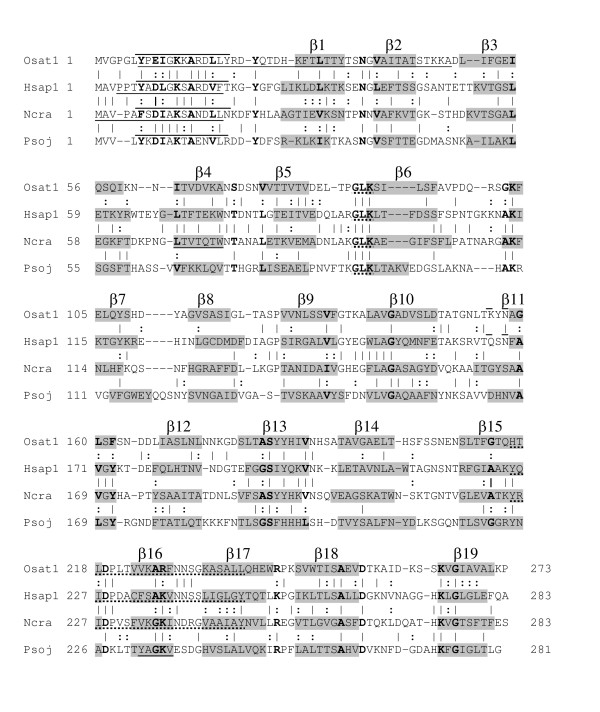
Alignment of representative porin sequences. Amino acid sequences from a plant (*O. sativa *VDAC1, Osat1,CAB82853), an animal (*H. sapiens *VDAC1, Hsap1, NP_003365), a fungus (*N. crassa*, Ncra, XP_323644) and a stramenopile (*P. sojae*, Psoj, C_1010023) were aligned using PRALINE, as described in Methods. Residues in grey background are predicted to reside in β-strands, overlined sequences in α-helical regions, and identical (|) and conserved (:) residues between adjacent pairs of sequences are indicated. Chemically-similar residues in all four representative sequences are shown in bold. The GLK motif (residues 87–89 of Osat1) and the existing eukaryotic porin motif (PS00558, residues 216–238 of Osat1) are indicated by dotted underlining. The VKAKV sequence (residues 224–228 of Osat1) is part of PS00558. Note that this motif is not found in the *P. sojae *VDAC sequence. Motifs, derived in this work, that are characteristic for plants (7–48 of Osat1), animals (4–18 of Hsap1) and fungi (1–16 and 68–74, separated by any 48–52 residues, Ncra) are underlined. A unique motif for the stramenopiles could not be discerned from the data available.

Although 19 β-strands are predicted by SSPRO, it is possible that all of these regions do no form β-strands. Regions including β1–β7 have been predicted by other algorithms (reviewed in [4]), but have not been experimentally tested. In the alignment of all sequences (see [Additional Files 3 and 4]), the region including β8 is not predicted to form a β-strand. However, when the plant or fungal sequences are aligned alone, a 6-residue β-strand is predicted in some sequences (residues 114–119 of *O. sativa *VDAC1, and 122–129 of *N. crassa *porin); in others this β-strand region is shorter (3–5 amino acid residues; for example see Fig. 5). A stretch of six residues is the minimum proposed to be sufficient for the β-strands in bacterial porins to span the thickness of a lipid bilayer [60]. In the animal sequences, β8 (residues 125–132 in *Homo sapiens *VDAC1) also contains sequence with α-helical propensity and therefore is unlikely to form a β-strand. Furthermore, this region contains a cysteine residue, which is absent from all β-strands in bacterial porins [60]. Therefore, if all porins have a common structure, the region including putative β8 may not form a β-strand. Another weak prediction is β11, which also has α-helical propensity in some of the sequences from all three groups. If these two putative β-strands are "disregarded", to maintain an even number of β-strands needed to complete the barrel [60], one other β-strand must be omitted. As discussed previously [4,16], although most algorithms predict a β-strand at the C-terminus of the protein, deletion analysis [15] suggests that this is not the case (see Fig. 4). Therefore, these data support a common, 16-strand pore structure, in which the interstrand loops and turns vary in size. However, it cannot be ruled out that porins from different organisms fold into barrels with different numbers of β-strands. In spite of the uncertainty in the number of predicted strands, the large data set examined strongly supports a common structural framework, in which the positions and lengths of the putative β-strands and the α-helix are extremely well conserved across all phyla investigated, and among all isoforms in a given organism.

In spite of the predicted structural similarities, there is a great deal of variation in the amino acid sequences that make up the putative strands and the intervening regions. To demonstrate this sequence diversity, VDAC sequences from one representative of the animals (*Homo sapiens *VDAC1), the fungi (*Neurospora crassa*), the plants (*Oryza sativa *VDAC1) and the stramenopiles (*Phytophthora sojae*) were aligned (Fig. 5). Considering identical and chemically similar residues, there are at most three positions that are related in three β-strands among all four sequences (β7, β11, β13), and in several strands there are no positions consistently occupied by chemically similar residues (β8, β14, β17).

### Signature Motifs for Eukaryotic Porins

The Prosite database carries one "signature" motif for eukaryotic porins (PS00558); this motif was derived from 30 animal, plant and fungal porins, including members of all major VDAC classes in these groups. To determine the universality of this motif, the current collection of porin sequences were analyzed. Of the 236 plant, animal and fungal sequences in the present analysis, 28 lack the complete C-terminal segment where this motif is found. The signature motif is not found in 92 of the remaining 208 sequences, indicating that it is not universal (Figs. 2 and 3), as noted by Wandrey *et al. *[40]. In particular, the motif is not detected in several animal sequences, such as zebrafish (DrerAAH654), nor in the limited number of stramenopile sequences available (Fig. 2).

The eukaryotic porin signature motif also contains the "VKAKV" sequence noted by Smith *et al. *[61] to be present in all VDAC sequences available at the time of their analysis. In our database, these residues were present in the following percentages of sequences: V (48.3), K (60.4), A (75.6), K (59.1), V (53.6). When complementary replacements are included, these numbers rise to V (81.6), K (70.5), A (84.5), K (98.7), V (85.7). This sequence resides in putative β16 (Fig. 5) and both lysine residues (K234 and K236) in *Saccharomyces cerevisiae *VDAC1 are required for membrane assembly of this protein [61]. The lack of strict conservation of these residues suggests that other features of this region also may contribute to membrane targeting or assembly.

Analysis of the aligned regions encompassing the eukaryotic signature motif did not reveal a motif common to all eukaryotic porins; this finding is not surprising given the low degree of sequence identity across the spectrum of sequences analyzed herein. Use of the PRATT algorithms ([62,63]) suggested that the N-terminal regions of the proteins contain sequences suitable for pattern analysis. Manual comparisons of the amino-terminal regions of the protein sequences visualized in GeneDoc [64] did reveal motifs common to most members of the largest phylogenetic groups: plants, fungi and animals (Fig. 5, Table 2). The limited number of stramenopile, charophyte and rhodophyte sequences did not allow identification of sequence patterns unique to this subgroup.

**Table 2:** Distribution of mitochondrial porin motifs in the sequences in the current analysis and public protein sequence databases.

Motif	Total Sequences Analyzed (this work)	Hits	Misses	Insufficient sequence data	Hits in Swiss-Prot TrEMBL and PDB ^a^	Expected random matches ^b^
Animal Motif ^c^	93 animal	76	4	13	81 (including 7 splice variants)	2.9 ^d ^(84)
Plant Motif ^e^	105 plant	90	11	4	43 (splice variants not identified)	2.1e-14
Fungal Motif ^f^	38 fungal	33	1	4	18 (splice variants not identified)	4.4 ^d ^(26)
Totals for this work	236	199	16	21	142	
PS00558 ^g^	93 animal	42	41	10	61 animal sequences	6.3e-02
PS00558	105 plant	59	35	11	30 plant sequences	as above
PS00558	38 fungal	20	15	3	11 fungal sequences	as above
PS00558	5 stramenopiles	0	1	4	0 stramenopile sequences	as above
Totals for PS00558	241	121	92	28	102 (94 without splice variants)	
GLK	93 animal	82	11 (2 GXK/R^h^)	0	28^i^	17478
GLK	105 plant	74	30 (11 GXK/R) 15 SXK	1	14	as above
GLK	38 fungal	30	8 (6 GXK/R)	0	2	as above
GLK	5 stramenopiles	5	0	0	0	as above
Totals for GLK	241	192	47 (19 GXK/R)	1	44 (of 50 VDAC sequences) ^j^	

^a ^sequences in UniProtKB/Swiss-Prot (release 50.6), UniProtKB/TrEMBL (release 33.6), PDB (14-Sep-2006) databases

^b^approximate number of expected random matches in Swiss-Prot release 41 (122564 sequences)

^c ^animal motif: <-X(0,30)-[PTSA]-X(1,2)-[YF]-X-[DE]-[ILVF]-[AG]-[KR]-X-[AST]-[KR]-[DE]-[ILV]-[FYST].; was not found in any plant or fungal VDAC sequences

^d ^expected random matches could not be calculated for constrained patterns; value was obtained using the unconstrained motif; in parentheses is the number of hits using the unconstrained motif

^e ^plant motif derived for Viridiplantae: [YF]-X-[DE]-[ILV]-G-[KR]-[KR]-[APST]-[KR]-D-[IL]-L-X-[KR]-D-[FHY]-X(4)-K-[FL]-[CNST]-X(4)-[ANST]-X(2)-G-X(2)-[FILV]-X-[ASTV]-[AST]-[AGS]-X(3)-[ADGNS].; was not found in any animal or fungal VDAC sequences

^f ^fungal motif: <-X(0,10)-[STIMLQ]-X(1,3)-[PL]-X(1,3)-[WYF]-X-[DEAG]-[ILV]-X-[RK]-X(3)-[DG]-X(0,1)-[ILV]-X(48,53)-[ILV]-X(2)-[ST]-Q-.96-[WL].; was not found in any plant or animal VDAC sequences

^g ^PS00558 (EUKARYOTIC_PORIN) [PROSITE (release 19.29)] motif: [YH]-x(2)-D-[SPCAD]-x-[STA]-x(3)-[TAG]-[KR]-[LIVMF]-[DNSTA]-[DNS]-x(4)-[GSTAN]-[LIVMA]-x-[LIVMY]

^h ^where X represents any amino acid

^i ^GXK/GXR included in total

^j ^the description (DE) filter VDAC was used due to the common occurrence of short sequences (G-L-K); this filter does not work on PDB sequence data, so only the Swiss-Prot and TrEMBL databases were searched

The fungi represent one of the smaller groups in terms of numbers of representative sequences [38], but the diversity in the group made it the most difficult in terms of identifying a consistent motif. The final sequence motif (Table 2) contains conserved residues in the N-terminal α-helix, and is anchored with a short conserved sequence around 50 residues away, which is part of the fourth putative β-strand. The only fungal VDAC sequence that did not contain this motif was that of *Botryotinia fuckeliana*. Updated sequence information may resolve this issue, as the *B. fuckeliana *VDAC sequence was derived from EST data. This fungal VDAC motif was somewhat more effective at identifying putative VDAC sequences in the public databases than the eukaryotic signature motif (Table 2).

The N-terminal animal VDAC motif derived in this work (Table 2) occurs once in each of 76 of the 80 animal VDAC sequences that had a complete N-terminal sequence in our database. Four sequences could not be accommodated without greatly reducing the specificity of the pattern, that of *Schistosoma japonicum*, and *Drosophila melanogaster *AAF53019 and AAF53018 and the ortholog from *D. pseudoobscura *(Dp-22; [47]). These *Drosophila *sequences diverge significantly from those of all porins in this study [see Additional File 2] and possess long N-terminal extensions [65,47]. The animal VDAC motif detects 81 sequences in the public databases, including seven spliceoforms. The position from the N-terminus of the protein was allowed to vary between 0 and 30 residues, to allow detection of human VDAC2 (P45880), in which the motif begins 24 residues from the predicted N-terminus.

The plant VDAC motif derived from this study (Table 2) was identified in all but eleven of the plant sequences used. All of the latter VDACs have related sequences in the N-terminal region, but their inclusion led to a consensus sequence with significantly reduced specificity. Both VDAC sequences from the green algae *Chlamydomonas reinhardtii *lack the motif described above. Within the land plants, it is absent from a small group of sequences located within a cluster of sequences united by node 2 (Fig. 2). Two closely related sequences within the cluster unified by node 5 (Fig. 2) also lack the motif. Three additional sequences shown in [Additional File 2] also lack the plant-derived VDAC motif, namely those derived from ESTs from a club moss *Selaginella moellendorffii*, and from *Pinus taeda *(gi|49625878), and from genomic DNA of *Sorghum bicolor *(TC94332). The plant motif is not contained within the VDAC sequences from organisms of the deeper branches of the tree: the red algae (*Galdieria sulphuraria *and *Cyanidioschyzon merolae*), and the Desmid *Chlosterium peracerosum-strigosum-littorale*. The plant VDAC motif detected 43 sequences in the public databases, and anchoring to the N-terminus was not necessary to maintain specificity.

Another highly conserved sequence in VDAC is the glycine-leucine-lysine (GLK) motif, which was initially suggested to be an ATP binding site [66]; replacement of the lysine residue with glutamate leads to porins that retain ATP binding [10], but are cation-selective, rather than anion selective in artificial bilayers [10,14].

The GLK sequence is present in the majority of sequences in the current survey (Table 2; Figs. 2 and 3). Variations containing chemically similar residues are also found (G-any-R, G-any-K, where "any" is any amino acid). In the fungi and animals, the VDAC sequences lacking this motif are scattered throughout the phylogenetic tree, and there is no apparent link between the presence of this motif and the eukaryotic signature sequence. In the plants there are two clusters of VDAC sequences in which GLK is replaced by a version of STK (Fig. 2, node 5). Also of note, all of the organisms that contain an STK-bearing isoform also have one with a GLK motif, suggesting perhaps a separation of function in the two types of isoforms.

VDAC 2.1 (STK) and VDAC 3.1 (no GLK or STK) isoforms from *Lotus japonicus *are able to complement a *Δpor1 *(VDAC1-less) strain of yeast (Table 1). In contrast, VDAC 1.3 does not complement in yeast, in spite of the fact that it possesses both a GLK and a eukaryotic signature motif (PS00558). However, further species or tissue-specific roles for these isoforms remain untested in plants, as does the pore-forming ability of these proteins. Taken together, the data suggest that the STK motif was either derived by a neutral event, or has been selected for as it was involved in a newly acquired, specialized function.

In terms of nucleotide differences in the corresponding coding sequences, the *Arabidopsis *isoforms provide an interesting example, as there is one with each of motif: GLK (AAM62480), GLR (NP_186777), STK (NP_201551) and SAK (NP_200557). Two transitions differentiate the AGG arginine codon in GLR from the AAA lysine codon in GLK. The SAK and STK containing two isoforms are found in the "ancient" cluster described by node 5. The STK sequence is encoded by TCC GCT AAA, while TCA ACA AAA encodes SAK. The SAK coding sequence differs from that encoding GLK (GGA CTG AAA) by three transversions and two transitions, while the difference between the STK and GLK sequences is five transversions and a transition – in both cases the lysine codon AAA is unchanged.

### Comparison of VDAC gene structures across a selection of species

**Figure 6:**
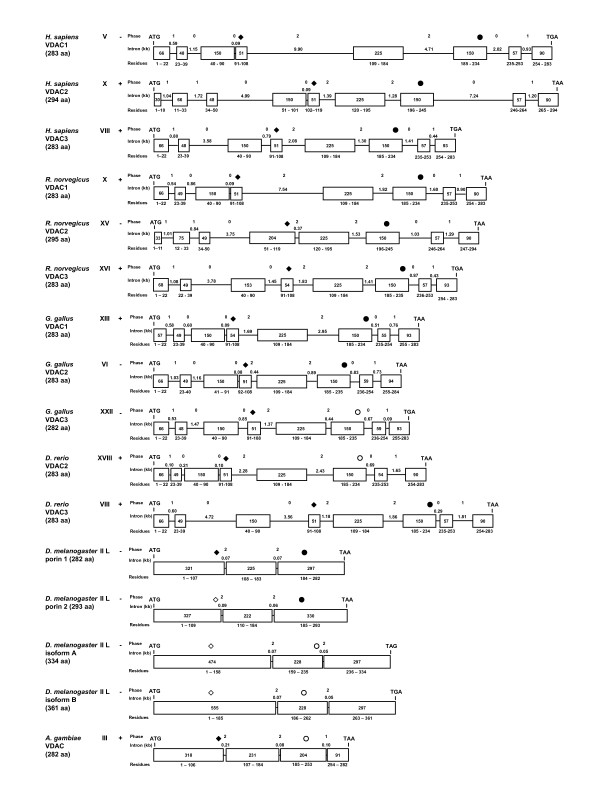
Comparison of animal mitochondrial porin gene structures. The exonic and intronic regions of mitochondrial porin open reading frames were mapped for a variety of organisms. The gene structure for all species was retrieved from the NCBI genome database using BLASTN or map viewer programs to analyze the ORF of each porin cDNA. Data were derived from the sequences described in the legend to Figure 8. Only regions of the gene containing ORFs are shown; chromosome number is indicated beside the species in Roman numerals and plus (+) or minus (-) strand reading frames are indicated beside chromosome number. Where multiple spliceoforms exist, as for human VDAC2, the form not listed as a splice variant was used. Lengths of exons in bp are indicated inside the boxes representing the exons; the numbers of the corresponding amino acid residues are indicated below the boxes. Lines indicate introns, the sizes of which are indicated in kb above the line; the intron phase (0, 1, or 2) is indicated directly above the intron size. Phase 0 introns do not interrupt codons, whereas phase 1 and phase 2 introns are positioned after the first and second nucleotides of the codon, respectively. The positions of the coding sequence for the GLK (diamonds) and the eukaryotic porin (circles) motifs are shown above the intron/exon map. Filled and open symbols indicate matches and imperfect matches to these motifs, respectively.

**Figure 7:**
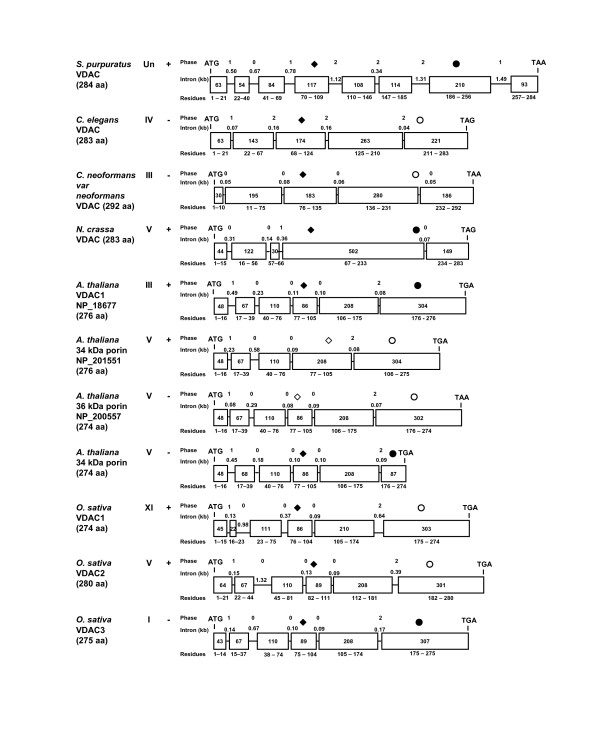
Comparison of fungal and plant mitochondrial porin gene structures. Symbols are as described for Figure 6, and data were derived from the sequences described in Figure 8.

**Figure 8:**
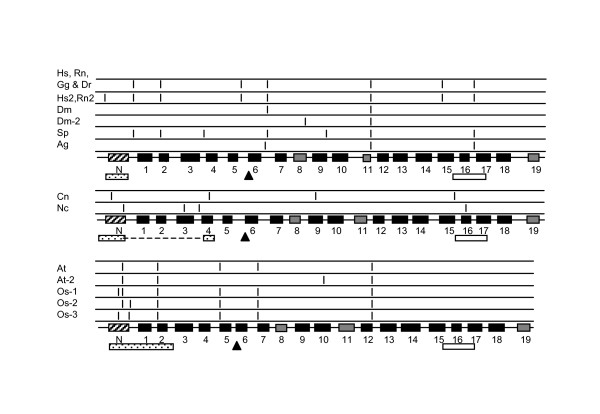
Intron placement with respect to coding sequences for conserved structural elements. Intron placement is indicated by vertical bars in the rows above the structural model for each crown group of organisms (see Fig. 4 for symbol descriptions). The positions of the GLK motif (triangles), the VDAC motifs identified in this study (stippled boxes) and the eukaryotic signature motif (open boxes) are indicated below the structural models. Note that the fungal VDAC motif is formed by two sequence elements; the intervening region is shown as a dotted line. Patterns of intron location that are found in several VDAC genes are shown only once. Animal sequences. Hs, *Homo sapiens *VDAC1 (NP_003365) and VDAC3 (NP_005653); Rn, *Rattus norvegicus *VDAC1 (NP_112643) and VDAC3 (NP_112645); Gg, *Gallus gallus *VDAC1 (TC10222), VDAC2 (NM_204741), VDAC3 (TC9741); Dr, *Danio rerio *accession numbers NM_199585 and BC065468; Hs2, *H. sapiens *VDAC2 (NP_003366), Rn2, *R. norvegicus *VDAC2 (NP_112644); Dm, *Drosophila melanogaster *VDAC1 (CG6647-PA), VDAC2 (NP_609462), and isoform B of CG31722 (AAF53018); Dm-2; *D. melanogaster *isoform A (AAF53019); Sp, *Strongylocentrotus purpuratus *(sea urchin, XM_775173); Ag, *Anopheles gambiae *(XM_318947). Middle panel: Fungal sequences. Cn, *Cryptococcus neoformans *(XM_569804.1); Nc, *Neurospora crassa *(XP_323644). Lower panel: Plant sequences. At, *Arabidopsis thaliana*, (NP_186777, NP_201551, NP_200057, NP_197013); At-2 *A. thaliana *(NP_190561); Os-1, *Oryza sativa *(CAB82853); Os-2, *O. sativa *(CAC80850); Os-3, *O. sativa *(CAC80851).

Given the shared predicted structural and primary sequence elements in mitochondrial porins, it was of interest to expand the initial analysis of porin genes (for example [67,68,47]). Therefore, twelve representative animal, plant, and fungal VDAC genes (including paralogs) were examined for the number of introns present, intron phasing, and the position of introns with respect to functional and structural motifs (Figs. 6, 7, 8). While this dataset is not large enough to derive statistically sound conclusions, many general trends were noted. The comparative VDAC gene maps show that the vertebrate VDAC open reading frames usually consist of 8 exons and 7 introns, a pattern also observed within the sea urchin (*Strongylocentrolus purpuratus*) VDAC gene. The *C. elegans *VDAC gene has 4 introns and the *Drosophila melanogaster *paralogs contain 2 introns. Thus, the number of introns appears to decrease and the introns get shorter as one goes from the vertebrates/echinoderms/nematodes to the arthropods. The plant VDAC paralogs examined typically had 4 to 5 short introns.

In general, introns were longest within the mammalian sequences sampled and shorter in the invertebrate, plant and fungal taxa (Figs. 6 and 7). This contrasts with the shorter exons noted within the chordate sequences, as exemplified by the human, rat, zebra fish and bird (*Gallus gallus*) VDAC genes in which exons range in size from 30 bp (human VDAC2) to 225 bps. There is a general trend among the vertebrate and echinoderm VDACs that, relative to the internal exons, the 5' terminal exons 1 and 2 are very short (30 to 75 bps), as are the 3' terminal exons 7 and 8 (55 and 93 bp, respectively). However, the echinoderm is an exception, with a relatively longer exon 7 (210 bp). In the chordates, exons 5 and 6 appear to be quite ancient as their size is conserved from zebra fish to humans.

Lawen *et al. *(2005) noted that the intron/exon junctions in human VDAC paralogs are conserved [69], *i.e. *a given intron disrupts the same position in a codon and therefore phase correlation exists. This correlation is also observed for the majority of introns in the chordate sequences examined (Fig. 6); the only exception is a single intron in rat VDAC2. Similar phase correlation was seen for the *Drosophila *isoforms (Fig. 6), and the plant sequences examined (Fig. 7). Intron phase correlation is viewed as evidence of non-random insertion of an intron and thus interpreted by some as the presence of an ancient intron [70]. Introns in phase 0 are viewed as old by some authors [71], as they do not disrupt a codon.

The fungal VDAC genes examined either lack introns (*S. cerevisiae VDAC1 *and *VDAC2, U. maydis*, data not shown) or show a bias towards phase 0 introns; overall we noted among the fungal VDAC paralogs displayed in Fig. 7 seven phase 0 introns and one phase 1 intron. The plant VDACs appear to have more phase 0 introns with a ratio of 20:7:7 for phase 0, 1 and 2 introns respectively. In contrast, there are 32 phase 0, 24 phase 1, and 22 phase 2 introns in vertebrate VDAC paralogs; thus there is not a strong bias towards any one category of intron. In contrast to this general trend, the *C. elegans *and Arthropod porin gene sequences appear to be devoid of phase 0 introns and the ratio of phase 1 to phase 2 introns is 2 to 13.

The coding sequences for the sequence motifs discussed above are also conserved with respect to exonic location (Fig. 8). In the animal sequences presented, the N-terminal motif is encoded by sequence within the first exon; the only exceptions are VDAC2 in *H. sapiens *and *R. norvegicus*. The 42-residue plant motif begins near residue six and includes the predicted α-helix. In contrast to the animal VDAC sequences, the coding region for the α-helix is disrupted by one or two introns. For the remainder of the plant motif, the coding region contains a single intron.

The fungal motif also begins about six residues from the N-terminus of the protein and the N-terminal portion of the motif (see above) spans 14–18 residues. In both fungal sequences in Fig. 7, this portion of the motif is encoded by exons 1 and 2. The C-terminal part of the motif is encoded by sequence including the junction between exons 2 and 3 in *C. neoformans*, and entirely by exon 4 in *N. crassa*. In the sequences examined, the entire coding sequence for the GLK motif is within a single exon. The coding sequence for the eukaryotic porin signature motif (PS00558) is split by an intron in all of the animal and fungal sequences in Fig. 8, except in *Drosophila*, while it is encoded by a single exon in the plant sequences.

### Intron location with respect to functional and structural motifs of VDAC

A more detailed examination of the position of introns with respect to the position of protein domains/or modules, such as the β-strands that are important components of the VDAC structure, showed that these modules appear to correlate with the position of introns. The introns in general do not interrupt β-strand modules or the GLK and eukaryotic porin motifs (Fig. 8). This observation is most pronounced in the vertebrate, sea urchin, *Drosophila *and plant VDAC genes; for example, there appears to be an intron conserved between β6 and β7 and between β11 and β12. In the metazoans, other introns are frequently located between the coding sequences for the N-terminal α-helix and the first β-strand module, between β1 and β2, and between β16 and β17.

Within the plant VDAC genes examined again there was a bias towards introns being located between β-strand modules (see Fig. 8). Introns were located between the coding sequences for β1 and β2, β4 and β5, β6 and β7, and β12 and β13. There is generally an intron within the coding region for the N-terminal α-helix as well. The limited data for *C. elegans *and the fungal VDAC genes did not allow conclusions to be made; as more complete sequences become available a more detailed examination of these groups of organisms can be carried out.

## Conclusion

Our phylogenetic analysis clearly shows that paralogs have appeared several times during the evolution of VDACs from the plants, metazoans, and even the fungi, suggesting that there are no "ancient" paralogs within this gene family. Secondary structure predictions are compatible with a common 16 β-strand framework for porin arrangement across the outer membrane. The eventual acquisition of detailed secondary structural information through x-ray crystallography or nuclear magnetic resonance spectroscopy (NMR) studies will allow proper assessment of the models developed in this study. This work also revealed sequence motifs characteristic of the members of the crown groups of organisms. The GLK (and homologous or analogous motifs) and the eukaryotic porin motifs in the Chordates tend to be in exons that appear to have changed little during the evolution of these metazoans. Further sequence data will allow further refinement of these motifs, and data from organisms such as stramenopiles, will allow more complete analysis of this poorly-represented group. Finally, intriguing connections between intron location and predicted structural features were observed. Our data support the notion that introns usually do not interrupt structural protein motifs, namely the predicted β-strands in this case. Among the metazoans two conserved porin sequence motifs (GLK motif and the "eukaryotic porin signature motif") are confined to potentially "old" exons, as suggested by the fact that these exons tend to be flanked by introns that usually are in the same phase (phase correlation). These observations concur with the concept of exon shuffling, wherein exons encode structural modules of proteins and that throughout the evolution the loss and gain of introns and the shuffling of exons via recombination events contributes towards the complexity of modern day proteomes [70,72]. The relevance of these observations will be come clearer as more genomic sequences and structural data become available for mitochondrial porins and other β-barrel, membrane spanning proteins.

## Methods

### Database searches for porin sequences

A variety of databases were searched in order to obtain VDAC sequences. Using a variety of different porin sequences as queries, GenBank was searched via blastp and tblastx against previously published VDAC sequences from related organisms. Porin sequences were also extracted from the genome sites listed in [Additional File 1]. Additional genome sequences were accessed via Munich Information Center for Protein Sequences [73], Broad Institute Fungal Genome Initiative [74] and The Institute for Genomic Research [75,76].

Over 280 potential/putative VDAC homologs were recovered from various databases. We also examined "chloroplast VDAC-like sequences", putative VDAC sequences from the slime molds, the causative agents of malarial diseases and microsporidia. However, for these we could not generate reliable alignments, with the exception of a plastid porin from *Zea mays *(TC249611). In particular, the putative VDAC sequences obtained from obligately intracellular parasites and the amitochondriate microsporidia appear to have evolved too quickly to yield meaningful comparisons. This is not surprising as these VDAC sequences likely have degenerated in these organisms or may have acquired or been co-opted for a new function. Thus, these VDAC-like sequences were excluded from our phylogenetic analysis.

### Phylogenetic Analysis

Sequences were aligned with the Clustal-X program [77] and manually refined with an alignment editor program (GeneDoc v2.5.010; [64]). The alignment was also refined with the online PRALINE multiple sequence alignment program [78,79] and this program was utilized for its secondary structure prediction capabilities [80]. The final data set comprised a total of 244 sequences [Additional File 3] and consisted of 372 nonambiguously aligned positions [Additional File 4]. A second data set was generated from the original alignment by extracting 141 sequences that represented the major groupings observed in the analysis of the larger data set. The smaller alignment was used in the Bayesian analysis that was very time consuming.

Phylogenetic trees based on Neighbor-Joining (NJ) were generated for the large (n = 244) and smaller data set (n = 141) using programs PROTDIST [JTT setting [81] and NEIGHBOR (NJ setting); these programs are contained within PHYLIP (Version 3.63, [82]). Bootstrap replicates (1000) were generated with SEQBOOT (PHYLIP) and evaluated with NJ analysis in combination with the CONSENSE program (PHYLIP) for obtaining a majority rule consensus tree.

Bayesian analysis was performed with the MRBAYES (version 3.1) program [83]. The necessary NEXUS file format for the alignment (input) file was generated with the file conversion option available within the DAMBE program [84]. The settings for MRBAYES were as follows: amino acid substitution model: mixed, gamma distribution with 4 gamma rate parameters. The Bayesian inference of phylogenies was initiated from a random starting tree and four chains were run simultaneously for 2 000 000 generations, with trees sampled every 100 generations. The first 25% of trees generated were discarded ("burn-in") and the remaining trees were used to compute the posterior probability values.

The phylogenetic trees and dendrogram presented were drawn with the TreeView program [85] using the PHYLIP or MRBAYES tree outfiles, and annotations were added to the figures with the aid of Corel Draw (Corel Corporation Limited).

### Secondary structure predictions

Secondary structure predictions were obtained using all plant, animal and fungal sequences aligned with PRALINE in individual groups, and as a whole set. Predictions were performed using Version 2 of SSPRO [86] following multiple alignment. Similar results (data not shown) were obtained with YASPIN [87]; some predicted β-strands were one or two residues longer in the YASPIN predictions, but otherwise their locations were the same. Based on analysis by EVA [88], the SSPRO algorithm results in a lower level of "confused" α and β-strand predictions than does YASPIN (BAD 3.0 vs 6.3), while the latter program generates higher percentages of correctly predicted strands compared to observed strands (QE%E60 72% for YASPIN vs 61% for SSPRO).

The conservation of amino acid sequence motifs was assessed on the aligned sequences using the column composition function of GeneDoc. The following groups of amino acids were considered chemically similar: (S, T), (I, L, V), (C, M), (K, R), (D, E), (F, Y, W), and (A, G).

## List of Abbreviations

VDAC, voltage-dependent anion-selective channel

nS, nanoSeimens

PTP, permeability transition pore

EST, expressed sequence tag

NJ, Neighbor-Joining

NMR, nuclear magnetic resonance spectroscopy

## Authors' contributions

MJY and DCB contributed equally to this work. MJY and DCB collected the sequence data and created the conceptual translations where necessary. MJY and GH carried out the phylogenetic analysis and DCB and DAC performed and analyzed the secondary structure predictions. DCB performed the intron/exon analysis. All authors participated in the design of the study, data analysis and the drafting of the manuscript and read and approved the final manuscript.

## Supplementary Material

Additional File 1 Eukaryotic Porin Sequences. The table provides descriptions of the sequences used for the analysis, their sources, their designations in the datasets in [Additional Files 3 and 4], and the narrow classification of the organisms that were the sources of the indicated porin sequences. Click here for file

Additional File 2 Phylogenetic estimate of the evolutionary history of 244 porin amino acid sequences. The phylogenetic tree is based on a NJ majority rule consensus tree constructed by analysing 1000 bootstrap replicates. Levels of confidence for nodes are only given if bootstrap support exceeded 70%. The chordate VDAC1, VDAC2 and VDAC3 groupings are indicated; the dashed portion of the line encompassing the VDAC3 group indicates putative VDAC3 molecules. Yeast VDAC1 and VDAC2 groupings are also shown. The putative VDAC2 of *C. glabrata *is highly diverged from the other yeast VDAC2s, but has been labelled VDAC2 as a strong candidate VDAC1 sequence was identified. Click here for file

Additional File 3 Mitochondrial porin sequence alignment in sequential (FASTA) format. The alignment of 244 mitochondrial porin sequences was obtained as described in Methods, and is presented in FASTA format. See [Additional File 1] for information regarding the source of individual sequences. Click here for file

Additional File 4 Mitochondrial porin sequence alignment in msf format. The alignment of 244 mitochondrial porin sequences was obtained as described in Methods, and is presented in msf format. See [Additional File 1] for information regarding the source of individual sequences. Click here for file
