# Supplementary material for: The evolutionary history of mitochondrial porins
Source: BMC Evol Biol. 2007 Feb 28;7:31. doi: 10.1186/1471-2148-7-31 (PMC1838418; doi:10.1186/1471-2148-7-31)
Supplement: Additional File 2 — Phylogenetic estimate of the evolutionary history of 244 porin amino acid sequences. The phylogenetic tree is based on a NJ majority rule consensus tree constructed by analysing 1000 bootstrap replicates. Levels of confidence for nodes are only given if bootstrap support exceeded 70%. The chordate VDAC1, VDAC2 and VDAC3 groupings are indicated; the dashed portion of the line encompassing the VDAC3 group indicates putative VDAC3 molecules. Yeast VDAC1 and VDAC2 groupings are also shown. The putative VDAC2 of C. glabrata is highly diverged from the other yeast VDAC2s, but has been labelled VDAC2 as a strong candidate VDAC1 sequence was identified. [file 1471-2148-7-31-S2.pdf]

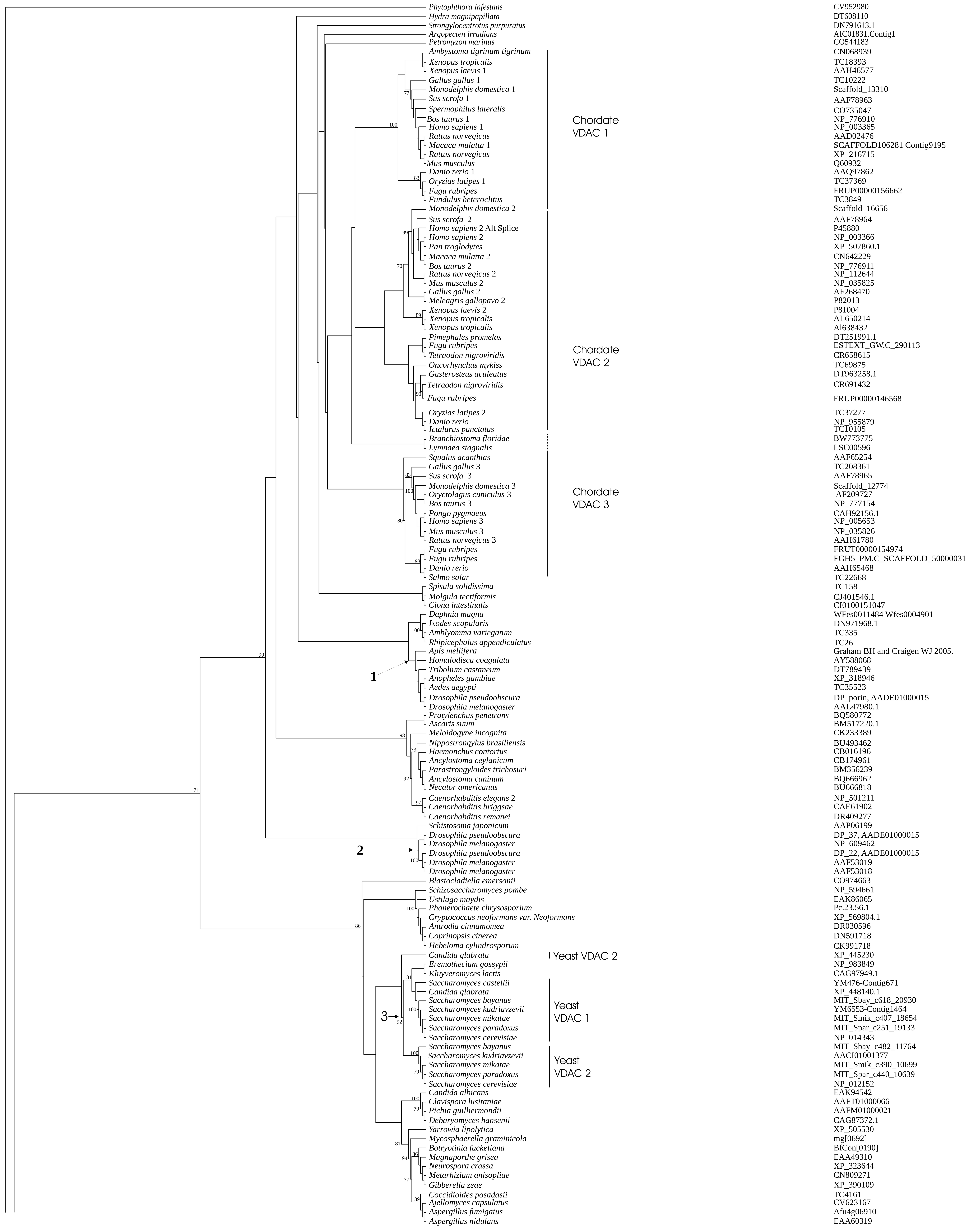

Phytophthora infestans CV952980
Hydra magnipapillata DT608110
Strongylocentrotus purpuratus DN791613.1
Argopecten irradians AIC01831.Contig1
Petromyzon marinus CO544183
Ambystoma tigrinum tigrinum CN068939
Xenopus tropicalis TC18393
Xenopus laevis 1 AAH46577
Gallus gallus 1 TC10222
Monodelphis domestica 1 Scaffold_13310
Sus scrofa 1 AAF78963
Spermophilus lateralis CO735047
Bos taurus 1 NP_776910
Homo sapiens 1 NP_003365
Rattus norvegicus AAD02476
Macaca mulatta 1 SCAFFOLD106281 Contig9195
Rattus norvegicus XP_216715
Mus musculus Q60932
Danio rerio 1 AAQ97862
Oryzias latipes 1 TC37369
Fugu rubripes FRUP00000156662
Fundulus heteroclitus TC3849
Monodelphis domestica 2 Scaffold_16656
Sus scrofa 2 AAF78964
Homo sapiens 2 Alt Splice P45880
Homo sapiens 2 NP_003366
Pan troglodytes XP_507860.1
Macaca mulatta 2 CN642229
Bos taurus 2 NP_776911
Rattus norvegicus 2 NP_112644
Mus musculus 2 NP_035825
Gallus gallus 2 AF268470
Meleagris gallopavo 2 P82013
Xenopus laevis 2 P81004
Xenopus tropicalis AL650214
Xenopus tropicalis Al638432
Pimephales promelas DT251991.1
Fugu rubripes ESTEXT_GW.C_290113
Tetraodon nigroviridis CR658615
Oncorhynchus mykiss TC69875
Gasterosteus aculeatus DT963258.1
Tetraodon nigroviridis CR691432
Fugu rubripes FRUP00000146568
Oryzias latipes 2 TC37277
Danio rerio NP_955879
Ictalurus punctatus TC10105
Branchiostoma floridae BW773775
Lymnaea stagnalis LSC00596
Squalus acanthias AAF65254
Gallus gallus 3 TC208361
Sus scrofa 3 AAF78965
Monodelphis domestica 3 Scaffold_12774
Oryctolagus cuniculus 3 AF209727
Bos taurus 3 NP_777154
Pongo pygmaeus CAH92156.1
Homo sapiens 3 NP_005653
Mus musculus 3 NP_035826
Rattus norvegicus 3 AAH61780
Fugu rubripes FRUT00000154974
Fugu rubripes FGH5_PM.C_SCAFFOLD_50000031
Danio rerio AAH65468
Salmo salar TC22668
Spisula solidissima TC158
Molgula tectiformis CJ401546.1
Ciona intestinalis CI0100151047
Daphnia magna WFes0011484 Wfes0004901
Ixodes scapularis DN971968.1
Amblyomma variegatum TC335
Rhipicephalus appendiculatus TC26
Apis mellifera Graham BH and Craigen WJ 2005.
Homalodisca coagulata AY588068
Tribolium castaneum DT789439
Anopheles gambiae XP_318946
Aedes aegypti TC35523
Drosophila pseudoobscura DP_porin, AADE01000015
Drosophila melanogaster AAL47980.1
Pratylenchus penetrans BQ580772
Ascaris suum BM517220.1
Meloidogyne incognita CK233389
Nippostrongylus brasiliensis BU493462
Haemonchus contortus CB016196
Ancylostoma ceylanicum CB174961
Parastrongyloides trichosuri BM356239
Ancylostoma caninum BQ666962
Necator americanus BU666818
Caenorhabditis elegans 2 NP_501211
Caenorhabditis briggsae CAE61902
Caenorhabditis remanei DR409277
Schistosoma japonicum AAP06199
Drosophila pseudoobscura DP_37, AADE01000015
Drosophila melanogaster NP_609462
Drosophila pseudoobscura DP_22, AADE01000015
Drosophila melanogaster AAF53019
Drosophila melanogaster AAF53018
Blastocladiella emersonii CO974663
Schizosaccharomyces pombe NP_594661
Ustilago maydis EAK86065
Phanerochaete chrysosporium Pc.23.56.1
Cryptococcus neoformans var. Neoformans XP_569804.1
Antrodia cinnamomea DR030596
Coprinopsis cinerea DN591718
Hebeloma cylindrosporum CK991718
Candida glabrata XP_445230
Eremothecium gossypii NP_983849
Kluyveromyces lactis CAG97949.1
Saccharomyces castellii YM476-Contig671
Candida glabrata XP_448140.1
Saccharomyces bayanus MIT_Sbay_c618_20930
Saccharomyces kudriavzevii YM6553-Contig1464
Saccharomyces mikatae MIT_Smik_c407_18654
Saccharomyces paradoxus MIT_Spar_c251_19133
Saccharomyces cerevisiae NP_014343
Saccharomyces bayanus MIT_Sbay_c482_11764
Saccharomyces kudriavzevii AACI01001377
Saccharomyces mikatae MIT_Smik_c390_10699
Saccharomyces paradoxus MIT_Spar_c440_10639
Saccharomyces cerevisiae NP_012152
Candida albicans EAK94542
Clavispora lusitaniae AAFT01000066
Pichia guilliermondii AAFM01000021
Debaryomyces hansenii CAG87372.1
Yarrowia lipolytica XP_505530
Mycosphaerella graminicola mg[0692]
Botryotinia fuckeliana BfCon[0190]
Magnaporthe grisea EAA49310
Neurospora crassa XP_323644
Metarhizium anisopliae CN809271
Gibberella zeae XP_390109
Coccidioides posadasii TC4161
Ajellomyces capsulatus CV623167
Aspergillus fumigatus Afu4g06910
Aspergillus nidulans EAA60319
Chordate VDAC 1
Chordate VDAC 2
Chordate VDAC 3
Yeast VDAC 2
Yeast VDAC 1
Yeast VDAC 2
1
2
3

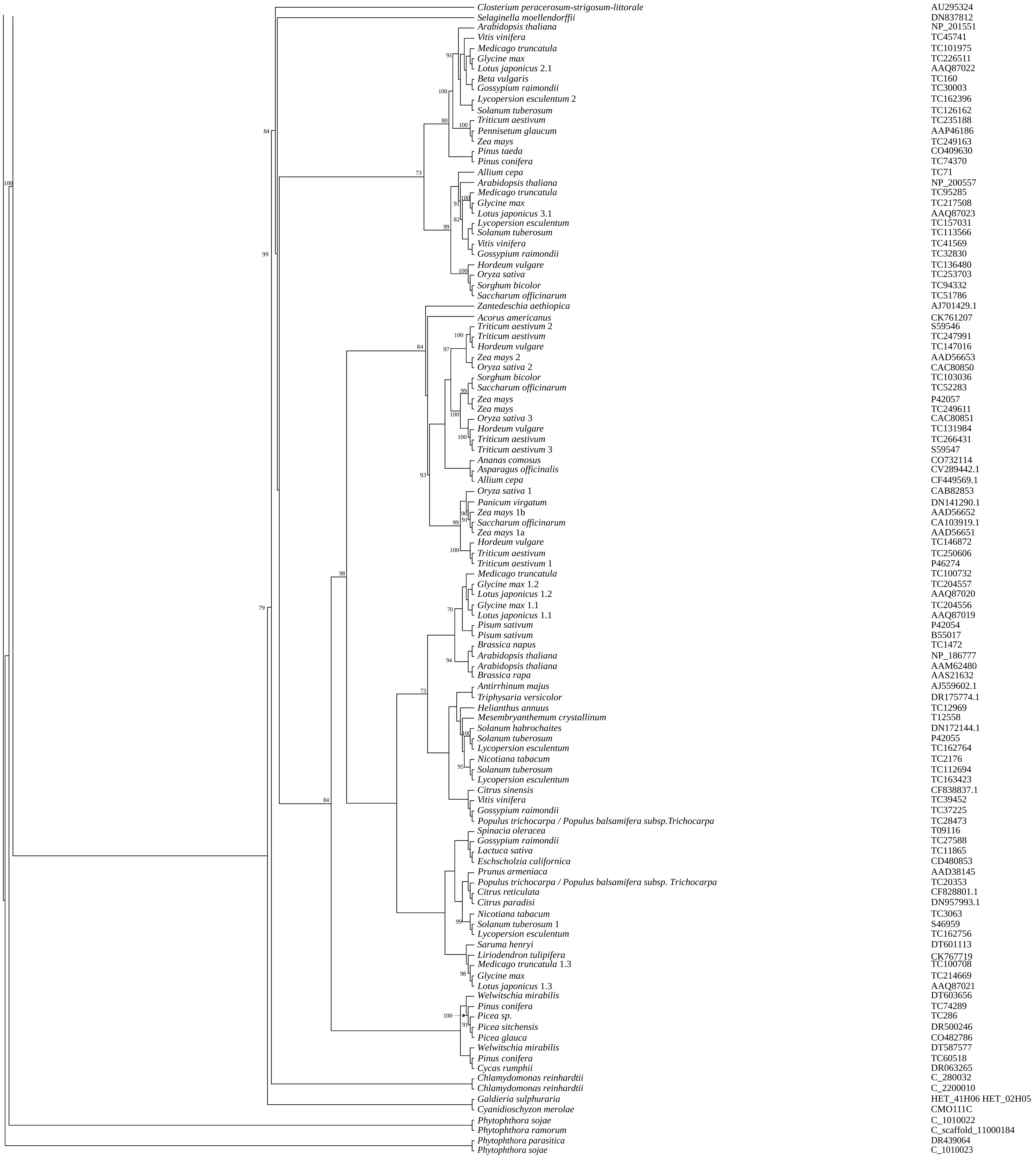

Closterium peracerosum-strigosum-littorale AU295324
Selaginella moellendorffii DN837812
Arabidopsis thaliana NP_201551
Vitis vinifera TC45741
Medicago truncatula TC101975
Glycine max TC226511
Lotus japonicus 2.1 AAQ87022
Beta vulgaris TC160
Gossypium raimondii TC30003
Lycopersion esculentum 2 TC162396
Solanum tuberosum TC126162
Triticum aestivum TC235188
Pennisetum glaucum AAP46186
Zea mays TC249163
Pinus taeda CO409630
Pinus conifera TC74370
Allium cepa TC71
Arabidopsis thaliana NP_200557
Medicago truncatula TC95285
Glycine max TC217508
Lotus japonicus 3.1 AAQ87023
Lycopersion esculentum TC157031
Solanum tuberosum TC113566
Vitis vinifera TC41569
Gossypium raimondii TC32830
Hordeum vulgare TC136480
Oryza sativa TC253703
Sorghum bicolor TC94332
Saccharum officinarum TC51786
Zantedeschia aethiopica AJ701429.1
Acorus americanus CK761207
Triticum aestivum 2 S59546
Triticum aestivum TC247991
Hordeum vulgare TC147016
Zea mays 2 AAD56653
Oryza sativa 2 CAC80850
Sorghum bicolor TC103036
Saccharum officinarum TC52283
Zea mays P42057
Zea mays TC249611
Oryza sativa 3 CAC80851
Hordeum vulgare TC131984
Triticum aestivum TC266431
Triticum aestivum 3 S59547
Ananas comosus CO732114
Asparagus officinalis CV289442.1
Allium cepa CF449569.1
Oryza sativa 1 CAB82853
Panicum virgatum DN141290.1
Zea mays 1b AAD56652
Saccharum officinarum CA103919.1
Zea mays 1a AAD56651
Hordeum vulgare TC146872
Triticum aestivum TC250606
Triticum aestivum 1 P46274
Medicago truncatula TC100732
Glycine max 1.2 TC204557
Lotus japonicus 1.2 AAQ87020
Glycine max 1.1 TC204556
Lotus japonicus 1.1 AAQ87019
Pisum sativum P42054
Pisum sativum B55017
Brassica napus TC1472
Arabidopsis thaliana NP_186777
Arabidopsis thaliana AAM62480
Brassica rapa AAS21632
Antirrhinum majus AJ559602.1
Triphysaria versicolor DR175774.1
Helianthus annuus TC12969
Mesembryanthemum crystallinum T12558
Solanum habrochaites DN172144.1
Solanum tuberosum P42055
Lycopersion esculentum TC162764
Nicotiana tabacum TC2176
Solanum tuberosum TC112694
Lycopersion esculentum TC163423
Citrus sinensis CF838837.1
Vitis vinifera TC39452
Gossypium raimondii TC37225
Populus trichocarpa / Populus balsamifera subsp.Trichocarpa TC28473
Spinacia oleracea T09116
Gossypium raimondii TC27588
Lactuca sativa TC11865
Eschscholzia californica CD480853
Prunus armeniaca AAD38145
Populus trichocarpa / Populus balsamifera subsp. Trichocarpa TC20353
Citrus reticulata CF828801.1
Citrus paradisi DN957993.1
Nicotiana tabacum TC3063
Solanum tuberosum 1 S46959
Lycopersion esculentum TC162756
Saruma henryi DT601113
Liriodendron tulipifera CK767719
Medicago truncatula 1.3 TC100708
Glycine max TC214669
Lotus japonicus 1.3 AAQ87021
Welwitschia mirabilis DT603656
Pinus conifera TC74289
Picea sp. TC286
Picea sitchensis DR500246
Picea glauca CO482786
Welwitschia mirabilis DT587577
Pinus conifera TC60518
Cycas rumphii DR063265
Chlamydomonas reinhardtii C_280032
Chlamydomonas reinhardtii C_2200010
Galdieria sulphuraria HET_41H06 HET_02H05
Cyanidioschyzon merolae CMO111C
Phytophthora sojae C_1010022
Phytophthora ramorum C_scaffold_11000184
Phytophthora parasitica DR439064
Phytophthora sojae C_1010023
